# Error Sources and Distinctness of Materials Parameters Obtained by THz-Time Domain Spectroscopy Using an Example of Oxidized Engine Oil

**DOI:** 10.3390/s18072087

**Published:** 2018-06-29

**Authors:** Mario Méndez Aller, Ali Mazin Abdul-Munaim, Dennis G. Watson, Sascha Preu

**Affiliations:** 1 Terahertz Systems Technology Group, Department of Electrical Engineering and Information Technology, Technische Universitat Darmstadt, Merckstr. 25, 64283 Darmstadt, Germany; aller@imp.tu-darmstadt.de; 2 Plant, Soil and Agricultural Systems, Southern Illinois University, MC 4415, Carbondale, IL 62901, USA; alimazin@siu.edu; 3 Department of Agricultural Machines and Equipment, College of Agriculture, Baghdad University, Baghdad 10071, Iraq

**Keywords:** Terahertz spectroscopy, optical path length, engine lubrication oil, error sources, thermal oxidation

## Abstract

Gasoline engine oil (SAE 5W-20) was subjected to thermal oxidization (TO) for four periods of time (0 h, 48 h, 96 h and 144 h) and exposed to THz-time domain spectroscopy (TDS) measurement. Error contributions from various error sources, such as repeatability errors, assembly errors of the probe volume and errors caused by the TDS system were evaluated with respect to discernibility and significance of measurement results. The most significant error source was due to modifications of the TDS setup, causing errors in the range of 0.13% of the refractive index for samples with a refractive index around 1.467 and a probe volume length between 5 and 15 mm at 1 THz. The absorption coefficient error was in the range of 8.49% for an absorption around 0.6 cm^−1^. While the average of measurements taken with different setup configurations did not yield significant differences for different TO times, a single, fixed setup would be able to discern all investigated oil species across the entire frequency range of 0.5–2.5 THz. The absorption coefficient measurement showed greater discernibility than the measurement of the refractive index.

## 1. Introduction

Terahertz (100 GHz–10 THz) sensor systems feature unique advantages in comparison to other spectral ranges, such as inspection of optically opaque substances [1]. Not only substances with narrow spectral features are being investigated but also broadband studies of the refractive index or the absorption coefficient vs. frequency [2]. A manifold of spectroscopic results have been obtained using broadband Terahertz time domain (TDS) spectroscopy, such as refractive indices and absorption coefficients of semiconductors [3] and various kinds of plastics [4]. While optical as well as microwave sensor concepts are highly developed with a manifold of calibration techniques and reference standards, there exists little metrology, with only a few studies on repeatability [5] or inter-system comparison and analysis of statistical and systematic error contributions [2,6,7,8,9], making comparison of results difficult.

In this paper, we investigated various error contributions to broadband TDS measurements of the absorption constant and the refractive index of liquid samples in a cuvette with a commercial system from Menlo Systems (C-fiber 1550) that is present in many laboratories. This included statistical errors, systematic errors due to the positioning of the sample and the sample size, as well as systematic errors due to variations in the TDS setup. We discuss scenarios where some of these errors can be suppressed, leading to improved repeatability under a controlled environment. As a study object, we selected thermally oxidized engine oil with oxidation times from 0 h to 144 h. This is a particularly interesting study object for both error analysis and scientific reasons. In terms of error analysis, the impact of the oxidation time on materials parameters of the oil is fairly faint, requiring high quality measurement results with low measurement errors. In terms of scientific impact, oxidation of engine oil is one of the major aging processes of engine oil. Operators of vehicles with internal combustion engines do not have access to comprehensive real-time analytical data of the engine lubricating oil. As a result, two scenarios are common. Operators unknowingly continue use of engine oil that is contaminated to a critical level, which can result in costly engine repairs. Alternatively, operators replace engine oil while it still has useful life; resulting in unnecessarily wasted engine oil. Both scenarios result in potential economic losses. In the absence of other contaminants, oxidation of oil during engine operation limits the useful life of engine oil.

The chemical interaction between oxygen atoms and hydrocarbon molecules in lubricating oil that breaks up hydrocarbon molecules is called oxidation. Oxidation leads to the formation of aldehydes and then acids [10], which decrease the lifespan of engine oil [11]. Higher oil temperatures accelerate oxidation [12]. Symptoms of engine oil oxidation are increased acidity [13], increased viscosity [14], gums [10] and sludge formation [15]. Additives in engine oil include antioxidants and detergents to counteract oxidation. When these additives are depleted, hydrocarbon molecules breakdown forming acidic compounds.

Terahertz (THz) time-domain spectroscopy (THz-TDS) has been applied to hydrocarbon fluids [16,17] and lubricating oil to distinguish among lubricating oil with and without additives [18], six grades of lubricating oil [19], oil base stock and additive [20], three different grades of gasoline engine oil [21], three different levels of water in diesel engine oil [22] and four different levels of fuel in gasoline engine oil [2].

## 2. Materials and Methods

### 2.1. Thermal Oxidation (TO) of Lubricating Oil

**Figure 1:**
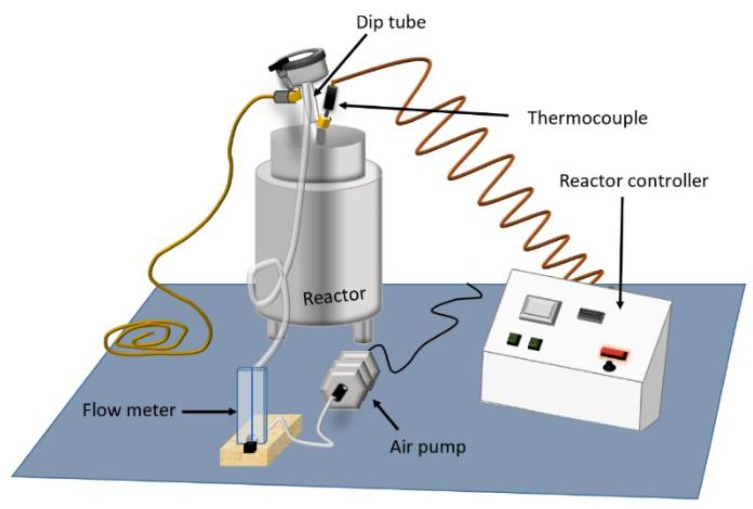
Equipment used to thermally oxidize engine oil.

Gasoline engine oil (SAE 5W20, API SN service category) was obtained from a local retailer (Carbondale, IL, USA) in a 4.73 L container. The SAE 5W20 grade is commonly specified by manufacturers for automobile engines. A means of thermally oxidizing oil was implemented based on an available reactor (model 4838, Parr Instrument Company, Moline, IL, USA; Figure 1). The reactor provided a stainless-steel cylinder for engine oil, temperature control, thermocouple well and input and exhaust ports for air. Ambient air was forced into the oil via an air pump and dip tube at the input port of the reactor. Air bubbled through the oil before exiting the exhaust port at the top of the cylinder. Regulated airflow of 1.0 L/min was provided by an acrylic flow meter (FL-2013, Omega Engineering, Inc., Norwalk, CT, USA). Plastic tubing (6 mm I.D × 10 mm O.D) directed the airflow from the pump to the inlet and from the outlet of the cylinder.

The temperature controller of the reactor was adjusted to reach a temperature of 180 °C and hold that temperature ±1 °C for the 48 h, 96 h and 144 h desired periods of oxidation [23,24]. Fresh engine oil at room temperature was used to pour 500 mL into the reactor cylinder. The reactor cylinder head was sealed and the TO process was initiated. After cooling, the oxidized oil was poured into a 500 mL amber glass container. A PTFE lined polypropylene cap was used to seal the glass container. Light exposure was limited by storing the oxidized sample in a closed cabinet.

The Technische Universität Darmstadt (Darmstadt, Germany) provided THz-TDS analysis of the oil samples. Approximately 50 mL each of fresh oil and oxidized oil samples were placed in separate 60 mL Boston round amber glass containers. Phenolic polycone caps were used to seal the containers before shipping to Technische Universität Darmstadt.

### 2.2. THz-TDS Spectrometer

**Figure 2:**
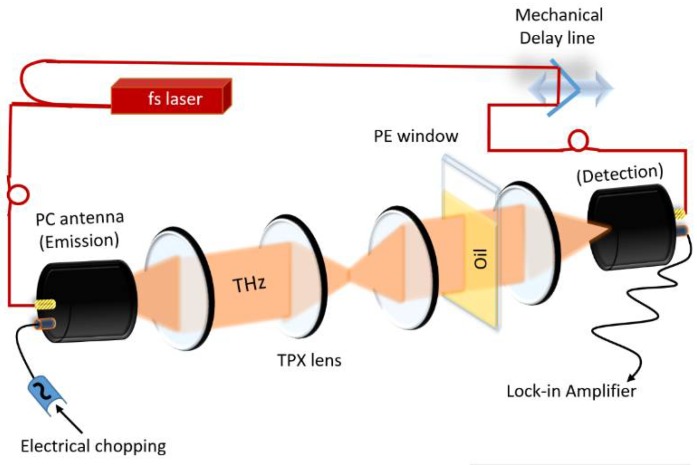
Depiction of a THz time-domain spectrometer.

The commercial THz-TDS spectrometer (Menlo Systems GmbH, Martinsried, Germany) used in this study had been detailed previously [2]. The system used a 1550 nm laser (pulse length < 90 fs) and fiber-coupled photoconductive antennas (Figure 2). Transmission configuration was used to measure refractive index and absorption coefficient of each oil sample in a cuvette. The cuvette was set up in the collimated THz beam in order to prevent shifting of the focal point at the receiver side. Polyethylene windows of 3 mm thickness were spaced 5–15 mm apart with metal spacers to form the cuvettes for this study. Echo reflection was used to measure the path length of an empty cuvette with a standard deviation of 10 µm. This variation was due to disassembly and reassembly variations and tilt variation when inserted in the spectrometer and were very close to the resolution limit of the system. In order to prevent cross contamination, the cuvettes were cleaned after disassembly with acetone to remove oil remainders, followed by an isopropanol cleaning step to remove acetone stains.

Spectra were averaged over 200 s or 500 s (averaged over 1200 or 3000 traces, respectively with 6 traces/s recorded), with a dynamic range of 20 dB for the most absorptive sample at 2.5 THz. THz-TDS results had a resolution of about 7 GHz in the frequency domain. Values in the range 0.5–2.5 THz were used for analysis. The measurement conditions are summarized as follows: temperature T = 23 °C ± 1 °C, normal pressure, the THz setup was purged with dry nitrogen to remove water vapor absorption lines, a time span of 80 ps, time step 33.4 fs, integration time/point 83.3 ms for the 200 s measurements and 208.3/point for the 500 s measurement, Hanning windowing for Fourier analysis (window size for most measurements 35 ps), THz 1/e² beam diameter 20 ± 1 mm and a cuvette window diameter of 35 mm × 35 mm.

The 3 mm polyethylene windows were thick enough to eliminate overlap of the main and reflected pulses. The phase shift of the Fourier transform of reference and sample measurements was extracted to calculate refractive index (*n*) and absorption coefficient (*α*) using the previously described equations [2,25],

(1) $$n_{S}\left(f\right)=1+\frac{c_{0}}{2\pi fd_{c}\;}\left(\varphi_{R}\left(f\right)-\varphi_{S}\left(f\right)\right)$$

(2) $$\alpha \left(f\right)=\;-\frac{2}{d_{c}}\ln\left(t\left(f\right)\frac{{\left(n_{s}\left(f\right)+n_{w}\right)}^{2}}{{\left(1+n_{w}\right)}^{2}n_{s}\left(f\right)}\right)$$

with *n_s_* the refractive index of the sample, *f* the frequency, $c_{0}$ the speed of light in vacuum, $d_{c}$ the probe volume length, *φ_S_*(*f*) and *φ_R_*(*f*) the Fourier phases of the sample and reference measurements, respectively, $n_{w}=1.535$ is the refractive index of the window and t(f) is the transmission ratio of the amplitude between sample and reference measurements at frequency *f*.

The following equations were used to approximate the error in refractive index (Δ*n*) and absorption coefficient (Δ*α*) based on the path length error, Δ*d*, with a standard deviation (95% confidence interval, CI) of 10 µm (19.6 µm) [2].

(3) $$\Delta n\approx \left(n-1\right)\frac{\Delta d}{d_{c}}$$

(4) $$\Delta \alpha \approx \left(\alpha -\frac{2}{d_{c}}\ln\left(\frac{{\left(n+n_{w}\right)}^{2}}{n{\left(1+n_{w}\right)}^{2}}\right)\right)\frac{\Delta d}{d_{c}}+2\left(\frac{2\left(n-1\right)\left(n+n_{w}\right)}{n{\left(1+n_{w}\right)}^{2}}+1\right)\frac{\Delta d}{d_{c}^{2}}$$

where $d_{c}$ is the measured thickness, Δd the thickness error, $n_{w}$ the refractive index of the windows and *n* and α are the measured values. Based on the 15 mm cuvette sample thickness, the 95% confidence interval (i.e., measurement error) for refractive index was calculated to be 0.04% (e.g., $\Delta n=5.9\times 10^{-4}$ for *n* ≈ 1.467 at 1 THz) and 0.52% (e.g., $\Delta \alpha =3.1\times 10^{-3}$ cm^−1^ for *α* ≈ 0.6 cm^−1^ at 1 THz) for absorption coefficient. According to Equation (3) an error due to inaccuracy of the measurement of the window material, Δ*n_W_*, has no influence on the accuracy of the refractive index because its influence is cancelled by the reference measurement of the empty cuvette. According to Equation (4) the absorption coefficient is weakly dependent on the window refractive index due to a modification of the reflection at the oil-window interface. Its influence is marginal, Δ*α* = 0.16/cm·Δ*n_W_*. An error Δ*n_W_*~10^−3^ results in an error of the absorption coefficient of 1.6 × 10^−4^ cm^−1^. that is, one order of magnitude smaller than the influence of the error in sample thickness. Therefore, we neglected any error in *n_W_*. Another source of a systematic error is the speed of light, *c*_0_, in Equation (1). For the visible, the refractive index of air had been determined to *n*_Air_ ≈ 1.00027 [26], dependent on environmental parameters like temperature, humidity and pressure. For dry nitrogen, that was used to purge the setup during measurements, we assume a refractive index even closer to 1 due to absence of water vapor. As we have no means for measuring the refractive index of dry nitrogen at THz frequencies to this accuracy, we assumed *n*_N2_ = 1, corresponding to a potential systematic offset of the cuvette length in the range of 4 µm at worst for the 15 mm cuvette. This is about 2.5 times smaller than the error due to disassembly and reassembly and would not influence the discriminability as it results in a systematic offset identical for all measurements.

## 3. Results

### 3.1. Measurement Error Analysis

Three different error sources of the TDS measurement were investigated: (i) statistical or repeatability errors caused by the measurement technique; (ii) systematic errors caused by the sample preparation, that is, due to errors in sample volume length by disassembly and reassembly. Disassembly and reassembly was necessary for cleaning purposes between recording THz spectra of samples with different oxidation times and (iii) systematic errors due to the setup. Though we used only one setup, data were recorded on different days with varying alignment of the TDS system, mimicking deviations in the setup, as well as two different averaging times (200 s and 500 s). All errors presented in this manuscript correspond to the 95% confidence interval of CI = 1.96σ, where σ is the standard deviation.

#### 3.1.1. Statistical Errors and Repeatability

For the purpose of statistical error analysis, the following measurement routine was used: (1) the cuvette was assembled; (2) a reference measurement was taken with the empty cuvette, averaging over 200 s; (3) the cuvette was filled with an oxidized oil species and (4) three consecutive TDS measurements were taken without disassembly and reassembly of the cuvette, again averaging over 200 s. The filled cuvette was taken out and reinserted into the setup between the consecutive measurements giving rise to a positioning error due to a slightly different angle of incidence of the THz wave on the cuvette. This slight variation gives rise to slight variations in refraction and therefore alters the path length of the THz wave within the cuvette which will be discussed in detail in Section 3.2.

For error data analysis, mean and 95% confidence interval (CI) at each frequency were calculated for *n* and *α* for each time period of oxidation for the three consecutive measurements, similar to prior studies [2,21,22]. Values were reported at 1.0 THz for comparison with other studies. Analysis of variance (ANOVA) was used to determine if there were significant differences (significance level = 0.05) among the *n* or *α* values of oxidation time for each frequency. Fisher’s least significant difference was used for pairwise comparison of oxidation times to determine which means were significantly different. Each measurement was considered a pseudo-replication for ANOVA [27]. SAS Enterprise Guide 7.1 software [28] was used for statistical analysis.

**Figure 3:**
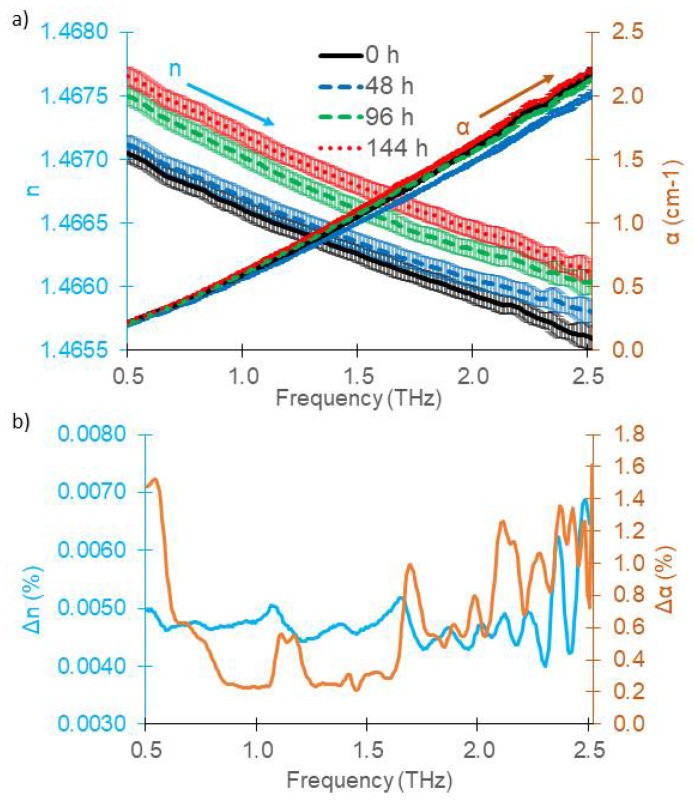
(**a**) Mean refractive index (left axis) and mean absorption coefficient (right axis) of gasoline engine oil (5W20) oxidized over four different times from three measurements of the 15 mm cuvette of gasoline engine oil with 95% confidence interval bars; (**b**) Relative values for the CI of the refractive index (left axis) and absorption coefficient (right axis).

Figure 3a shows the recorded refractive index for different TO times including the 95% confidence interval bars for *d*_c_ = 15 mm. The general trend of the refractive index is a strictly monotonous decrease with increasing frequency for all TO times. The refractive index of different TO times differs only in the 4th digit. The total error due to cuvette positioning and due to statistical errors of the measurement technique caused fairly frequency-independent errors in the refractive index (e.g., Δ*n*_rep_ = 7 × 10^−5^ (0.0048%) at 1.0 THz). On a first glance, the data suggest that the different TO times are well discriminable and that *n* increased with TO time. The refractive indices were 1.4666, 1.4667, 1.4670 and 1.4672 at 1.0 THz respectively for thermal oxidation times of 0 h, 48 h, 96 h and 144 h. However, the cuvette needed to be disassembled for cleaning between measurements of oils with different TO times, giving rise to additional systematic errors caused by preparation of the cuvette which are discussed in the next section. Figure 3 therefore does not allow for conclusions on discernibility of the TO times by THz measurements.

Figure 3a also depicts the absorption coefficient. The *α* values ranged from 0.2 at 0.5 THz to 2.2 at 2.5 THz with a repeatability error less than Δ*α*_rep_ = 1.4 × 10^−3^ cm^−1^ (0.22%) at 1.0 THz. Measurements of oils with different TO times seem well discernible. TO leads first to a reduction of absorption coefficient, indicating destruction of polar components in the lubricant, while it strongly increases again for TO times longer than 48 h.

Figure 3b shows the relative values for the 95% confidence interval for both the refractive index and the absorption coefficient, taking all *N* = 12 measurements into account. The CI then calculates to $\mathrm{CI}=1.96\sqrt{\frac{\sum_{i=1}^{N}{\left(x_{i}-\bar{x}\right)}^{2}}{N-4}}$, where $x_{i}$ is either the *i*^th^ refractive index measurement or *i*^th^ absorption coefficient measurement and $\bar{x}$ is the mean value for the respective TO time. The term *N*-4 in the denominator takes into account that the four mean values are statistically dependent on the measurement values.

Up to about 2.3 THz, the CI of the refractive index remains below 0.0051%. The CI for the absorption coefficient remains below 1.5% for the whole frequency range and shows some peak-like structures. These are most likely attributed to faint remainders of water vapor in the system. Most reliable frequency ranges for discernibility are between 0.85 and 1.05 THz and 1.25–1.6 THz, where the CI drops below 0.32%.

Mean *α* values at 1.0 THz were 0.5989, 0.5657, 0.6099 and 0.6330 cm^−1^, respectively for TO times of 0 h, 48 h, 96 h and 144 h. Highly significant differences (*p* < 0.0001) were found among thermal oxidation times across the 0.5–2.5 THz range. However, also these values are prone to systematic errors by dis- and reassembly and do not allow for judging on discernibility.

#### 3.1.2. Systematic Errors Due to Sample Preparation

Disassembly and reassembly of the cuvette lead to variations in probe volume and THz interaction lengths or an unwanted angle between the two windows (e.g., by tightness of the locking screws of the PE windows). Errors in probe volume length or sample thickness are very common. Often, the probe volume length is determined by a mechanical measurement (e.g., by a caliper) with a measurement inaccuracy in the range of several tens of µm to 100 µm. For this study, cuvette volume length was directly probed with a TDS measurement. The empty cuvette was inserted into the setup and the main transmission peak as well as the first round trip were recorded. From their temporal displacement, Δ*t* = 2*n*_N2_*d*_c_/*c*_0_, the length of the probe volume in the cuvette, *d*_c_, was determined, yielding a systematic error due to disassembly and reassembly of the 15 mm cuvette of *σ* = 10 µm ± 4 µm (CI: 19.6 µm) assuming *n*_N2_ = 1. This is much more accurate than a mechanical length measurement with a caliper. The absolute error of the refractive index of the oils with different TO times due to disassembly and reassembly of the cuvette according to Equation (3) was Δ*n*_Sys1_ = 5.95 × 10^−4^ (0.04%) at 1 THz and below 0.0405% over the whole range up to 2.3 THz, that is, about an order of magnitude larger than Δ*n*_Rep_.

**Figure 4:**
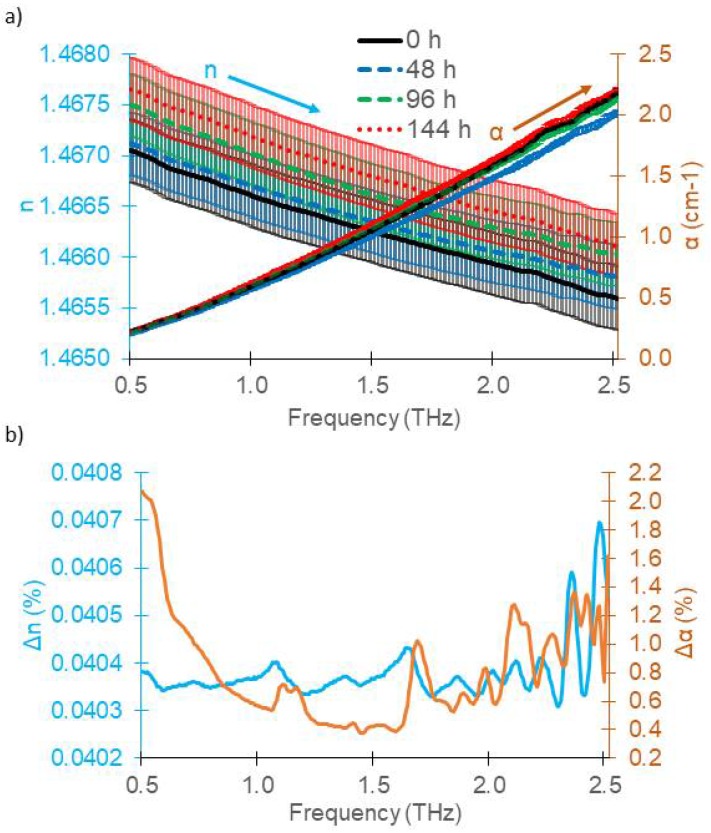
(**a**) Mean refractive index and absorption coefficient of gasoline engine oil (5W20) oxidized over four different times from three measurements of the 15 mm cuvette; (**b**) 95% confidence intervals for *n* and *α*, taking the CI of the probe volume thickness into account.

As shown in Figure 4a,b, the additional error due to sample preparation resulted in error bars greater than 95% confidence intervals (Figure 3b), that is, the refractive index measurement does not allow to discern different TO times by a THz measurement.

For the absorption coefficient, the error due to probe volume length fluctuations with a confidence interval of 19.6 µm lead to an error of Δ*α*_d_ = 3.1 × 10^−3^ cm^−1^ at 1 THz according to Equation (4), yielding a total error of $\Delta \alpha =\sqrt{\Delta \alpha_{d}^{2}+\Delta \alpha_{rep}^{2}}=3.4\times 10^{-3}$ cm^−1^ (0.56% for *α* = 0.6 cm^−1^ at 1 THz). The measurements of the absorption coefficient remained discernible. The absorption index can therefore be considered as more robust for data analysis and sample comparison for samples with an absorption coefficient as low as about 0.5 cm^−1^.

#### 3.1.3. Systematic Errors Due to the Spectroscopy System

**Figure 5:**
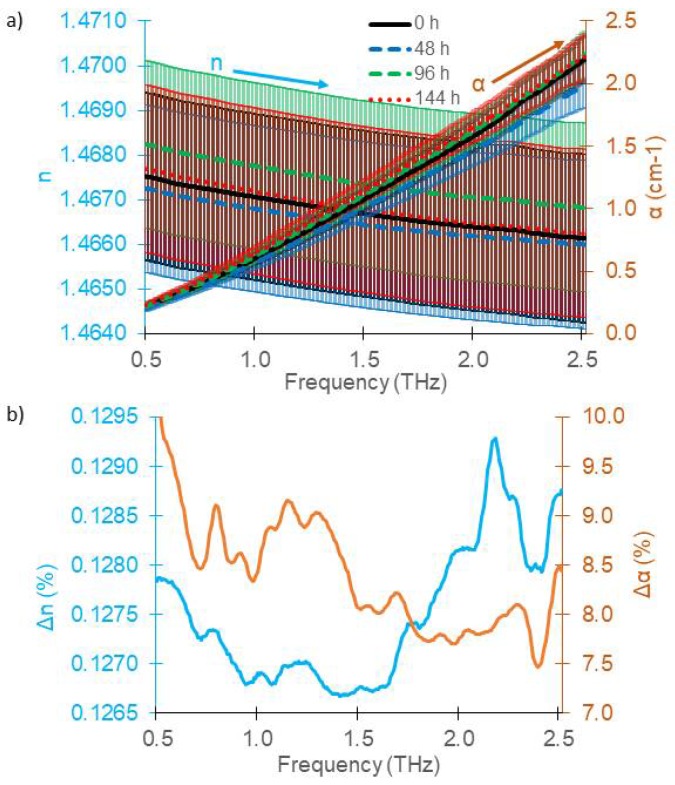
(**a**) Refractive index and absorption coefficient with 95% confidence intervals from 5 different measurement setups; (**b**) 95% confidence interval with *N* = 5 different setups.

Cases I and II take mainly relative errors into account. Comparing results obtained with a single setup may allow for discerning reliably different grades or contamination levels of oils but these values may contain systematic offsets that disallow providing any judgement on the absolute error of the refractive index and absorption coefficient. Comparing results from different research groups on fresh gasoline engine oil of the same brand and grade but different batch [2,18] already shows deviations in mean refractive index in the range of 0.0056 at 1 THz (0.38%). Though fluctuations in oil quality and production processes may have contributed to these differences, it is generally accepted that differences in the setup and measurement technique cause additional measurement errors of the same order of magnitude. In order to assess the errors caused by the measurement technique, we characterized the oils with different TO times with three different cuvette thicknesses (5 mm, 10 mm and 15 mm). Further, the measurements with the 10 mm and the 15 mm cuvettes were repeated with a different alignment of the THz path. The 15 mm measurement was recorded at 200 s averaging, while the other measurements were recorded at 500 s averaging. For obtaining the frequency spectra from the time domain measurements, different window sizes, ranging from 20 to 40 ps, for the Fourier transformation were further used. Figure 5a shows the refractive index and the absorption coefficient while Figure 5b shows the CI. The CI was calculated using all data of the *N* = 5 independent measurements with *M* = 4 different TO times according to $CI=\frac{1}{M}\sum_{i=1}^{M}CI_{i}$ with $I_{i}=1.96\sqrt{\frac{\sum_{j=1}^{N}{\left(x_{ji}-{\bar{x}}_{i}\right)}^{2}}{N-1}}$ , where x=n or x=α, respectively. Figure 5a shows the refractive index and the absorption coefficient. Neither *n* nor *α* are discernible any more. Figure 5b shows the CI vs. frequency. The CI for the refractive index at 1 THz was in the range of Δ*n*_Sys2_ = 1.9 × 10^−3^ (0.13%), that is, 3 times larger than if the same setup (case II) was used and in excellent agreement with measurement errors obtained in [6] where standard deviations around 10^−3^ (CI = 2 × 10^−3^) were reported. The CI of the absorption index was Δ*α*_Sys2_ = 0.052 cm^−1^ at 1 THz (8.49%, averaged over all TO times), that is, 15 times larger than in case II causing a small overlap of the confidence intervals. 

### 3.2. Discussion of Error Sources

**Figure 6:**
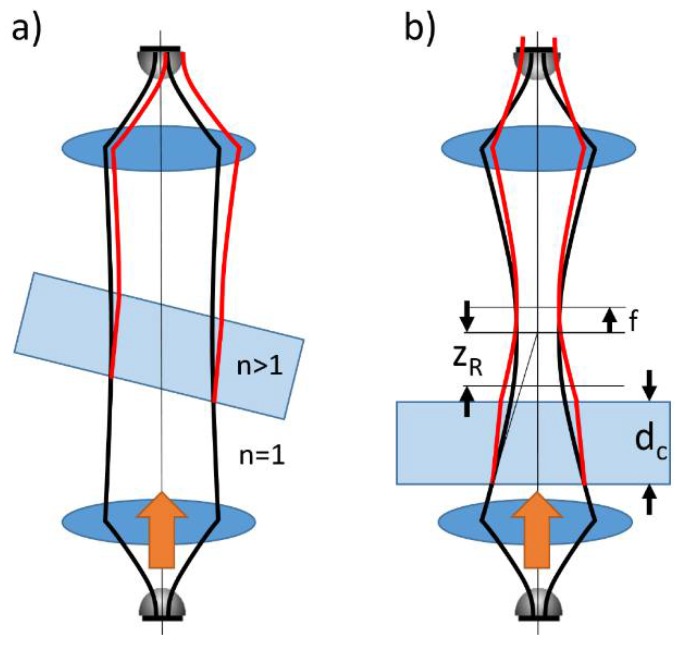
Examples of beam propagation errors caused by a sample. Black: beam propagation without sample. Red: deviation of the beam propagation with sample inserted. (**a**) beam walk off due to a small inclination angle of the sample and (**b**) focusing error due to imperfect beam collimation or by a very long sample with a thickness longer than the Rayleigh length, *d*_c_ > *z*_R_, causing a shift of the Gaussian beam waist in the measurement path (indicated by f) and a focal shift at the receiver. Both cases not only cause pointing or focusing errors, they also alter the beam profile, leading to reduced transmission form source to receiver. Deflection by the silicon lenses is not shown here.

Most errors were due to an error of THz path length within the probe volume. Statistical and repeatability errors (case I) were due to slight errors in the incidence angle of the THz wave on the cuvette after reinsertion as illustrated in Figure 6a. While perpendicularly incident waves traveled straight through the probe volume, samples mounted with a slight angle caused refraction of the THz wave, resulting in a slightly longer optical path length within the sample. Further, for long samples, the wave propagates away from the optical axis which may cause pointing errors from source to receiver, resulting in an overestimation of the absorption. In case study I, we showed that such effects remain considerably small, only affecting the 4th or 5th digit of the refractive index and the 3rd to 4th digit of the absorption index for a cuvette thickness of 15 mm at a refractive index around 1.467 and absorption around 0.5–2 /cm^−1^ as long as care is taken to properly align the samples.

The systematic error in case II caused by sample preparation strongly affected the discernibility of results. Errors in THz path length, *d*_c_, due to dis- and reassembly are of statistical nature. They can only be prevented if the same cuvette was used without dis- and reassembly for cleaning. Such errors would therefore not be present in a fixed, permanently filled cuvette for oil quality monitoring in a car engine. Such a fixed setup might not even experience repeatability errors from case I as long as other error sources, like thermal expansion of the probe volume can be prevented.

The systematic error in case III caused by the system as such is a major issue for comparing results from different laboratories. There is a manifold of reasons for deviations of the measurement results, mostly related to different or imperfect alignment of the THz beam (see Figure 6a,b) and references [6,7]). These cause, for instance, longer THz path lengths than expected from the geometry of the sample, or shifts of the focal point, causing lower recorded amplitudes than actually transmitted through the sample. While such effects do cause an offset error of the refractive index and of the absorption, they usually do not affect the discernibility of measurements taken in the very same setup: The systematic offset error will be similar and always have the same sign for the same setup. Similar to case II, a fixed setup would not be affected by this error source. 

We used the same software for calculating the materials parameters from all obtained TDS spectra. Further systematic errors may originate from different data evaluation routines. Even for excellent data evaluation routines, a typical problem arises at low frequencies (<200 GHz typically), where the error of Fourier transformation of the time domain data strongly increases. The finite measurement window represents a truncation of the data, typically yielding a (small) DC or low frequency offset of the time domain data. The Fourier transformation to the frequency domain then suffers from a divergence at *f*→0 which may affect results up to 100–200 GHz. This effect was partly taken into account in case (III). Also, different windowing methods and analysis techniques play a role. Discussing these goes beyond the aim of this paper. Data evaluation issues will again strongly affect the comparability of results obtained in different laboratories but for a single, fixed setup with a fixed data evaluation routine, these will mainly result in a systematic offset, not destroying discernibility of results. For a detailed discussion of data evaluation routines, we refer to [29,30].

Repeatability errors (case I) increase with sample length or larger refractive index due to a larger displacement of the optical axis (Figure 6a). Comparability errors discussed in case III also increase if strongly different sample lengths are investigated in different setup configurations causing focal displacement errors (Figure 6a,b). Errors discussed in case II generally decrease with probe volume length. Longer path lengths only negligibly increase the (optical) thickness error and beam propagation error but strongly increase the measurement accuracy, in particular for the absorption coefficient. The error according to Equations (3) and (4) decreases as the mounting error, Δ*d* and should not depend on cuvette thickness as long as cuvettes have comparable stiffness and do not flex. Therefore, the ratio Δ*d*/*d*_c_ in Equations (3) and (4) decreases. Even if ratio Δ*d*/*d*_c_ remained constant, the error in absorption coefficient is dominated by the last term in Equation (4) for the samples investigated in this paper. This term scales as Δ*d*/*d*_c_^2^, further reducing the error in absorption coefficient at large probe volume lengths.

However, there are limitations on the maximum length: firstly, the setup might not accommodate extremely long samples. Secondly, only a collimated beam (plane wave) trespasses the sample without changing shape. The THz beam can be considered as a Gaussian beam rather than a plane wave. Gaussian beams cannot be collimated over infinite distances, the beam waist is a function of propagation length with respect to the Rayleigh length, *z*_R_. Since the sample features a refractive index *n* > 1 different from that of the reference measurement, that is, air (*n* ≈ 1), introduction of the sample increases the optical path length in the setup and, hence, modifies the Gaussian beam propagation and beam waist position as illustrated in Figure 6b. This leads to focusing errors, reducing the power transmitted from source to receiver and an overestimation of the absorption. Therefore, the sample needs to be thin vs. the Rayleigh length of the Gaussian beam. A particularly bad sample position for thick samples is in the vicinity of a sharp focal point, where the divergence angle of the beam is large, as for example, in ref. [8], which resulted in a fairly large standard deviation error of the refractive index Δn~10^−2^, being an order of magnitude worse than reported in this manuscript. For the samples studied here, we did not see a major influence of the sample thickness, proving that the probe volume length is much smaller than the Rayleigh length of the collimated beam and the system was well aligned. 

Considering all investigated error sources, the absorption coefficient of weakly to moderately lossy materials such as the investigated oils is a much more reliable predictor than the refractive index although its relative error appears larger. Due to Lambert-Beer law, changes in the absorption coefficient result in an exponential change of the transmission, overcompensating the worse relative error in our case. Table 1 summarizes the results on errors found in this study.

## 4. Conclusions

The refractive index and absorption coefficient of gasoline engine oil (SAE 5W-20) subjected to thermal oxidization for four periods of time (0 h, 48 h, 96 h and 144 h) was spectroscopied by Terahertz time domain spectroscopy and analyzed for various sources of measurement errors. Repeatability errors of a fixed setup resulted in errors in the refractive index of Δ*n*_rep_ = 5 × 10^−5^ for a refractive index around 1.467 and an error in absorption coefficient of <1 × 10^−3^ cm^−1^ for an absorption around 0.6 cm^−1^ at 1 THz for a cuvette length of 15 mm. Sample preparation caused slight variations in the probe volume length. At 1 THz, the refractive index experienced an error of Δ*n*_Sys1_ = 3 × 10^−4^ and the absorption coefficient experienced an error of Δ*α*_Sys1_ = 1.59 × 10^−3^ cm^−1^. While the refractive index error caused an overlap of the 95% confidence intervals, the error in *α* is much smaller than the differences in absorption of all investigated TO times. Mean absorption values at 1.0 THz were 0.5989, 0.5657 0.6099 and 0.6330 cm^−1^, respectively, for TO times of 0 h, 48 h, 96 h and 144 h. Finally, variations in the setup configuration were examined by using cuvette lengths between 5 and 15 mm and variations in the alignment of the setup, averaging time of spectra and window size. Different measurement configurations caused confidence intervals in the refractive index in the third digit (Δ*n*_Sys2_ = 1.9 × 10^−3^ = 0.13%) and in the second digit of the absorption coefficient (Δ*α*_Sys2_ = 0.052 cm^−1^ = 8.49%) for all investigated samples. While this error source causes severe problems for comparing results from different laboratories, it usually causes a systematic offset rather than a statistical error and therefore does not affect the discernibility if a single, fixed setup is used, where the error is at least an order of magnitude smaller. We therefore conclude that THz-TDS demonstrated good potential for distinguishing differences in engine oil caused by thermal oxidation. Based on this study, continued exploration of THz-TDS for engine oil contaminants is warranted to determine the extent of the THz-TDS potential to distinguish other engine oil contaminants.

## Figures and Tables

**Table 1:** 95% confidence level for the three different cases in relative representation at 1 THz.

	Repeatability Error (Case I)	Total Error Incl. Sample Preparation (Case II)	Inter-System Comparability Error (Case III)
Δn at 1 THz	0.0048%	0.04%	0.13%
Δ*α* at 1 THz	0.22%	0.56%	8.49%
