# Erratum: Mendez Aller, M. et al. Error Sources and Distinctness of Materials Parameters Obtained by THz-Time Domain Spectroscopy Using an Example of Oxidized Engine Oil. Sensors. *Sensors* 2018, *18*, 2087

**DOI:** 10.3390/s18124459

**Published:** 2018-12-16

**Authors:** Mario Méndez Aller, Ali Mazin Abdul-Munaim, Dennis G. Watson, Sascha Preu

**Affiliations:** 1 Terahertz Devices and Systems, Department of Electrical Engineering and Information Technology, Technische Universitat Darmstadt, Merckstr. 25, 64283 Darmstadt, Germany; aller@imp.tu-darmstadt.de; 2 Plant, Soil and Agricultural Systems, Southern Illinois University, MC 4415, Carbondale, IL 62901, USA; alimazin@siu.edu; 3 Department of Agricultural Machines and Equipment, College of Agricultural Engineering Sciences, University of Baghdad, Baghdad 10071, Iraq

The authors wish to correct the affiliation of co-author Ali Mazin Abdul-Munaim, due to name changes of which he was unaware during his leave of absence. The correct affiliation is Department of Agricultural Machines and Equipment, College of Agricultural Engineering Sciences, University of Baghdad, Baghdad, Iraq. The authors would like to apologize for any inconvenience caused to the readers by these changes.
